# A novel mouse model for septic arthritis induced by *Pseudomonas aeruginosa*

**DOI:** 10.1038/s41598-019-53434-5

**Published:** 2019-11-14

**Authors:** Tao Jin, Majd Mohammad, Zhicheng Hu, Ying Fei, Edward R. B. Moore, Rille Pullerits, Abukar Ali

**Affiliations:** 1 Department of Rheumatology and Inflammation Research, Institute of Medicine, Sahlgrenska Academy at University of Gothenburg, Göteborg, Sweden; 2 Department of Rheumatology, Sahlgrenska University Hospital, Göteborg, Sweden; 3 Department of Microbiology and Immunology, The Affiliated Hospital of Guizhou Medical University, Guiyang, China; 4 Department of Infectious Diseases, Institute of Biomedicine, Sahlgrenska Academy, University of Gothenburg, Göteborg, Sweden; 5 Culture Collection University of Gothenburg (CCUG), Sahlgrenska Academy, University of Gothenburg, Göteborg, Sweden; 6 Centre for Antibiotic Resistance Research (CARe), University of Gothenburg, Göteborg, Sweden; 7 Department of Clinical Immunology and Transfusion Medicine, Sahlgrenska University Hospital, Göteborg, Sweden

**Keywords:** Bacterial host response, Bacterial infection

## Abstract

Septic arthritis is one of the most aggressive joint diseases. Although caused predominantly by *S. aureus*, Gram-negative bacteria, *Pseudomonas aeruginosa* among them, account for a significant percentage of the causal agents of septic arthritis. However, septic arthritis caused by *P. aeruginosa* has not been studied thus far, due to lack of an animal model. NMRI mice were inoculated with different doses of *P. aeruginosa*. The clinical course of septic arthritis and radiological changes of joints were examined. Furthermore, the host molecular and cellular mechanisms involved in *P. aeruginosa*-induced septic arthritis were investigated. Inoculation of mice with *P. aeruginosa* caused septic arthritis in a dose-dependent manner. Neutrophil depletion led to higher mortality and more severe joint destruction (*p* < 0.01). In contrast, monocyte depletion resulted in higher mortality (*p* < 0.05) but similar arthritis severity compared to controls. Mice depleted of CD4+ T-cells inoculated with *P. aeruginosa* displayed less severe bone damage (*p* < 0.05). For the first time, a mouse model for *P. aeruginosa* septic arthritis is presented. Our data demonstrate that neutrophils play a protective role in *P. aeruginosa* septic arthritis. Monocytes/macrophages, on the other hand, are only essential in preventing *P. aeruginosa*-induced mortality. Finally, CD4+ T-cells are pathogenic in *P. aeruginosa* septic arthritis.

## Introduction

Septic arthritis, remains one of the most aggressive joint diseases^1^. It is characterized by rapidly progressing joint and cartilage destruction. The prevalence rate of septic arthritis is approximately 6 cases per 100,000 in the general population and much higher in rheumatoid arthritis patients^1^. Furthermore, mortality rates of septic arthritis remain quite high; with 5–20% of the patients succumbing to the disease^1,2^ and as many as half of surviving patients suffering from permanent joint dysfunction^1,2^. *Staphylococcus aureus (S. aureus)*, a Gram-positive bacterium, is the most common cause of septic arthritis^1,2^. However, Gram-negative bacteria, with *P. aeruginosa* among them, account for a significant percentage as the causal agent of septic arthritis^3^. The prevalence of *P. aeruginosa* septic arthritis is especially higher in immune-compromised patients, as well as in intravenous drug abusers^3^. *P. aeruginosa*, an opportunistic pathogen, is responsible for a wide range of acute and chronic infections, including nosocomial infection^4^, and is the main cause of mortality among cystic fibrosis patients^5^. Septic arthritis caused by Gram-negative bacteria is associated with poorer prognosis compared to septic arthritis caused by Gram-positive bacteria. The mortality rate is higher (25% vs 6%, respectively) and only 20% of patients with septic arthritis triggered by Gram-negative bacteria regain joint function^6,7^.

During the past few decades, our laboratory has been at the forefront in *S. aureus* septic arthritis research. Using a unique animal model of *S. aureus* septic arthritis, we have been able to characterize the virulence factors of the bacterium as well as the responses of the host immune cells and cytokines^8–12^. However, there are virtually no studies regarding *P. aeruginosa*-induced septic arthritis, mainly due to the lack of animal model for the disease. We describe here a murine septic arthritis model induced by haematogenous spread of *P. aeruginosa* that mimics human septic arthritis and, furthermore, we characterize the cellular and molecular pathways involved in the pathogenesis of this disease.

## Results

### *P. aeruginosa* induces septic arthritis in mice in a dose-dependent manner

To determine whether *P. aeruginosa* could induce septic arthritis, mice were injected intravenously (i.v.) with different doses of *P. aeruginosa* and followed for up to 10 days.

**Figure 1:**
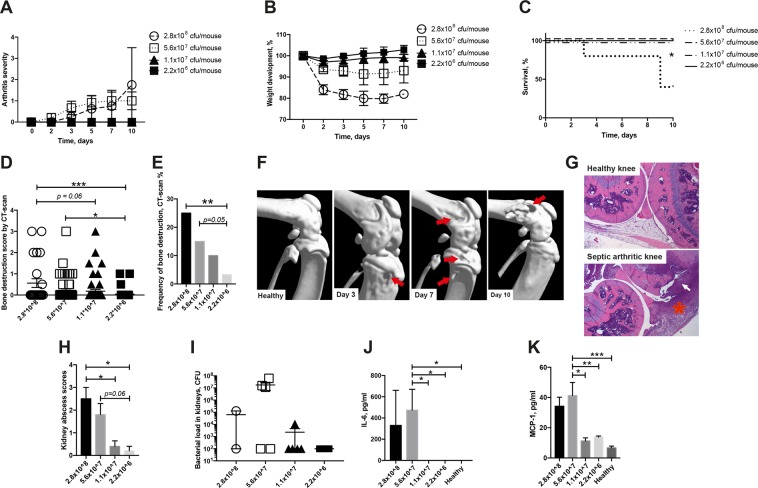
*P. aeruginosa* induces septic arthritis in mice. Naval Medical Research Institute (NMRI) mice were inoculated with different doses of *Pseudomonas aeruginosa* (*P. aeruginosa*) (2.2 × 10^6^–2.8 × 10^8^ colony-forming units [CFU]/mouse) and followed for up to 10 days. The severity (**A**) of arthritis in the mice were observed for 10 days post-infection. Y values represent measurements from surviving mice only for the respective days. Changes in body weight expressed as percentages of the initial body weight (**B**) and cumulative survival (**C**) of the mice. Cumulative bone destruction scores (**D**) and frequency of bone destruction (**E**) of the joints from all 4 limbs of NMRI mice as assessed by micro-computed tomography scan. A representative micro-computed tomography images (**F**) of an intact knee joint from healthy NMRI mouse and destroyed knee joints from NMRI mice with septic arthritis on day 3, 7 and 10 post-infection. The arrows indicate bone destruction. Representative photomicrographs (**G)** of histologically intact knee joint from a healthy NMRI mouse (upper panel) and of a heavily inflamed knee joint with severe bone and cartilage destruction from NMRI mouse with septic arthritis inoculated with *P. aeruginosa* (lower panel), stained with hematoxylin and eosin. Original magnification, ×10. The asterisk indicates heavily inflamed synovium. Abscess scores of the kidneys from the mice sacrificed 10 days post-infection (**H**) and, bacterial load of *P. aeruginosa* in kidneys of the mice (**I**). Levels of the pro-inflammatory cytokine Interleukin 6 (IL-6) (**J**) and chemokine monocyte chemoattractant protein 1 (MCP-1) (**K**) in serum were determined after termination of the experiment on day 10 post-infection. The data from 2 independent experiments were pooled, (n = 5–11/group). Statistical evaluations were performed using the Mann–Whitney U test (**A,B,D,H–K**), Log-rank Mantel cox (**C**) and Fisher’s exact test (**E**). Data are expressed as mean values ± SEM. *p < 0.05; **p < 0.01, ***p < 0.001.

The mice receiving the relatively low dose (≤1.1 × 10^7^ colony forming units [CFU]/mouse) of *P. aeruginosa* did not exhibit any signs of clinical arthritis whereas clinical arthritis was observed in mice receiving the higher doses (≥5.6 × 10^7^ CFU/mouse) of *P. aeruginosa*. However, there were no significant differences between these groups (Fig. 1A). The main reason behind this might be the high mortality rate in the mice receiving the highest dose of *P. aeruginosa*.

Using micro-computed tomography (CT), we could evaluate joints that are unable to be assessed clinically, e.g. knees, hips, elbows and shoulders. In these samples, we observed significantly more severe bone destruction (*p* < 0.001) as well as higher frequencies of bone erosions (*p* < 0.01) in the mice receiving the higher doses of *P. aeruginosa* compared to mice receiving lower doses (Fig. 1D–F). The subgroup analyses of bone destruction are shown in Supplementary File (see Supplementary Tables S1 and S2).

A dose-dependent pattern regarding weight loss among mice receiving different doses of *P. aeruginosa* was also observed (Fig. 1B). Mice inoculated with higher doses of *P. aeruginosa* lost significantly more weight (*p* < 0.05) during the course of the experiment compared to mice receiving lower doses of bacteria.

Significantly more mice succumbed to *P. aeruginosa* in the group receiving the highest dose of bacteria compared to all other groups (*p* < 0.05) (Fig. 1C). In fact, 60% of the mice in the highest bacterial dose group had died by day 9.

A dose-dependent pattern regarding the kidney abscess score was also observed: mice inoculated with the highest dose of *P. aeruginosa* developed more macroscopic kidney abscesses compared to those inoculated with lower doses of the bacteria (*p* < 0.05) (Fig. 1H). Although not statistically significant, dose-dependent tendencies for higher CFU counts were observed in the kidneys of mice receiving the higher doses of *P. aeruginosa* (5.6 × 10^7^ CFU/mouse, Fig. 1I).

Interestingly, a dose-dependent pattern regarding the serum IL-6 and MCP-1 levels were observed in the mice. Mice receiving higher doses of *P. aeruginosa* (5.6 × 10^7^ CFU/mouse) had significantly higher levels of both IL-6 (*p* < 0.05) and MCP-1 (*p* < 0.001) serum compared to mice receiving lower doses (Fig. 1J,K).

The definitive diagnosis of septic arthritis is the recovery of bacteria in the joints. To this end, joints from *P. aeruginosa* infected mice were collected, homogenized and plated. Five out of six mice had at least one joint that was positive for CFU counts.

To study which cytokines are responsible for the onset of *P. aeruginosa*-induced septic arthritis, supernatants collected from joint homogenates of *P. aeruginosa* infected mice (7 × 10^7^ CFU/mouse) were compared to homogenates from healthy mice. Significantly elevated levels of TNF-α and IL-6 were observed among the *P. aeruginosa* infected mice compared to healthy mice (see Supplementary Fig. S1). However, no differences with regard to IL-10 and MCP-1 were observed between the groups (see Supplementary Fig. S1).

### Neutrophils are protective against *P. aeruginosa-*induced septic arthritis

**Figure 2:**
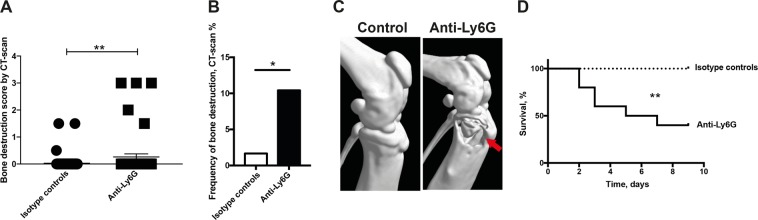
Neutrophils are protective against *P. aeruginosa* induced septic arthritis. Naval Medical Research Institute (NMRI) mice received intraperitoneally (i.p) a volume of 200 μl (400 μg/injection) of Anti-Ly6G in order to deplete neutrophils on days −1 and +1 post-infection with *Pseudomonas aeruginosa* (*P. aeruginosa*) (2.0–5.6 × 10^6^ colony forming units [CFU]/mouse). The same volumes and concentration of injected isotype control antibody served as controls (n = 10/group). Cumulative bone destruction scores (**A**) and frequency of bone destruction (**B**) of *P. aeruginosa* infected NMRI mice on day 10 post-infection as assessed by micro-computed tomography scan. A representative micro-computed tomography image (**C**) of an intact knee from healthy NMRI mouse treated with isotype control (left panel) and heavily destroyed knee joint from NMRI mouse with septic arthritis depleted of neutrophils (right panel). The arrow indicates bone destruction. The cumulative survival of the mice (**D**) during the course of the experiment was also assessed. Statistical evaluations were performed using the Mann–Whitney U test (**A**), Fisher’s exact test (**B**) and Log-rank Mantel cox (**D**). *p < 0.05; **p < 0.01.

To understand the role of neutrophils in *P. aeruginosa-*induced septic arthritis, mice were either depleted of neutrophils by anti-Ly6G antibodies or received isotype control antibodies. Mice lacking neutrophils exhibited significantly higher severity (*p* < 0.01) as well as higher frequency of bone erosions (*p* < 0.05) when infected by *P. aeruginosa* compared to isotype controls (Fig. 2A–C). The subgroup analyses of bone destruction are shown in Supplementary File (see Supplementary Table S3).

Neutrophils are absolutely crucial for the defence against *P. aeruginosa-*induced mortality, as mice depleted of neutrophils had significantly higher mortality (*p* < 0.01), compared to their isotype controls (Fig. 2D). Only 40% of the mice depleted of neutrophils survived until termination of the experiment while the survival rate for the controls was 100%.

### Monocytes/macrophages are essential in protection against *P. aeruginosa*-induced mortality

**Figure 3:**
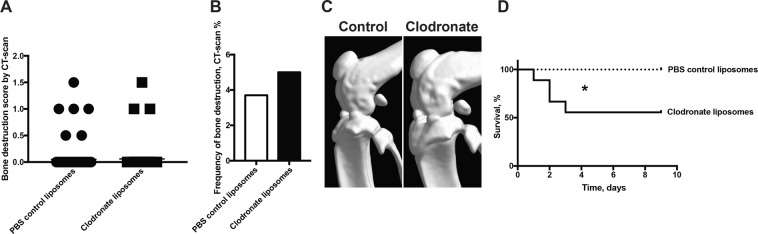
Macrophages are essential against *P. aeruginosa* induced mortality. Naval Medical Research Institute (NMRI) mice received a volume of 200 μl of clodronate liposomes intravenously in order to deplete monocytes/macrophages on days −1 and +1 post-infection with *Pseudomonas aeruginosa* (*P. aeruginosa*) (2.0–5.6 × 10^6^ colony forming units [CFU]/mouse). The same volumes of phosphate-buffered saline control liposomes were injected as control (n = 9/group). Cumulative bone destruction scores (**A**) and frequency of bone destruction (**B**) of *P. aeruginosa* infected mice on day 10 post-infection as assessed by micro-computed tomography scan. A representative micro-computed tomography image (**C**) of an intact knee from healthy NMRI mouse treated with PBS control liposomes (left panel) and an intact knee from healthy mouse depleted of monocytes/macrophages (right panel). The cumulative survival of the mice (**D**) during the course of the experiment was also assessed. Statistical evaluations were performed using the Mann–Whitney U test (**A**), Fisher’s exact test (**B**) and Log-rank mantel cox (**D**) *p < 0.05.

We proceeded further and investigated the role of monocytes/macrophages in *P. aeruginosa*-induced septic arthritis by depleting blood monocytes using clodronate liposomes. In contrast to mice depleted of neutrophils, the lack of monocytes/macrophages did not have any impact on the severity or on the frequency of bone destruction induced by *P. aeruginosa* compared to control mice (Fig. 3A–C). The subgroup analyses of bone destruction are shown in Supplementary File (see Supplementary Table S4). However, monocytes/macrophages, just like neutrophils, are essential for the protection of the host against *P. aeruginosa*-induced mortality. Mice lacking macrophages had significantly higher mortality (*p* < 0.05) compared to their controls (Fig. 3D), with approximately 45% of the macrophage-depleted mice succumbing to the disease compared to none from the control mice.

### Septic arthritis induced by *P. aeruginosa* is mediated through CD4 but not CD8 T-cells

**Figure 4:**
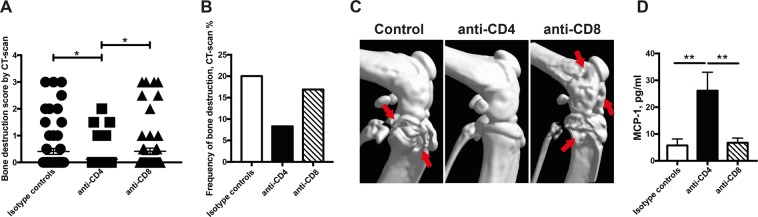
Septic arthritis induced by *P. aeruginosa* is mediated through CD4 but not CD8 T-cells. Naval Medical Research Institute (NMRI) mice received a volume of 200 μl intraperitoneally (400 μg/injection) of anti-CD4 or anti-CD8 in order to deplete CD4 and CD8 T-cells on days −1, +3 and +7 post-infection with *Pseudomonas aeruginosa* (*P. aeruginosa*) (1.2 × 10^7^ colony forming units [CFU]/mouse). The same volumes and concentration of injected isotype control antibodies served as control (n = 5/group). Cumulative bone destruction scores (**A**) and frequency of bone destruction (**B**) of *P. aeruginosa* infected NMRI mice on day 10 post-infection as assessed by micro-computed tomography scan. A representative micro-computed tomography image (**C**) of a heavily destroyed knee joint from NMRI mouse with septic arthritis treated with isotype control (left panel), intact knee from mouse depleted of CD4-T-cells (middle panel) and heavily destroyed knee joint from mouse with septic arthritis and depleted of CD8-Tcells (right panel). The arrows indicate bone destruction. Serum levels of monocyte chemoattractant protein 1 (MCP-1) were assessed (**D**). Statistical evaluations were performed using the Mann–Whitney U test (**A,D**) and Fisher’s exact test (**B**). Data are expressed as mean values ± SEM.*p < 0.05; **p < 0.01.

Next, we investigated the role of T-cells in *P. aeruginosa*-induced septic arthritis by selectively depleting either CD4 or CD8 T-cells in the mice. Interestingly, the severity of bone destruction was significantly lower in mice depleted of CD4 T-cells (*p* < 0.05) compared to mice treated with isotype control antibody (Fig. 4A,C). This is in line with our previous study^13^ that demonstrated CD4 T-cells to be arthritogenic in staphylococcal septic arthritis. CD8 T-cells do not seem to have any impact in *P. aeruginosa*-induced septic arthritis since mice depleted of CD8 T-cells had similar severity and frequency of bone destruction as control mice (Fig. 4A–C). The subgroup analyses of bone destruction are shown in Supplementary File (see Supplementary Table S5). Intriguingly, mice depleted of CD4 T-cells displayed significantly higher levels of serum MCP-1 (*p* < 0.01) compared to both isotype controls as well as mice lacking CD8 T-cells during *P. aeruginosa* i.v. infection (Fig. 4D).

### IL-1 does not play a crucial role in *P. aeruginosa*-induced septic arthritis

**Figure 5:**
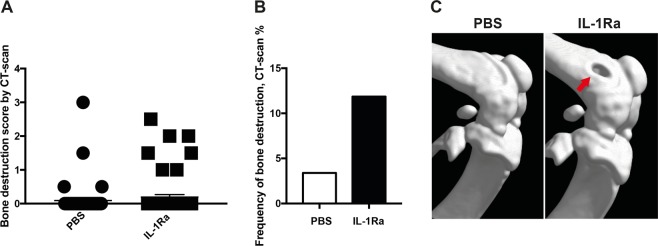
IL-1 does not play a crucial role in *P. aeruginosa* induced septic arthritis. Naval Medical Research Institute (NMRI) mice inoculated with *Pseudomonas aeruginosa* (*P. aeruginosa*) (2 × 10^6^ colony forming units [CFU]/mouse) were intraperitoneally treated with anakinra (IL-1Ra, 400 μg/mouse in 100 μl of PBS, n = 5), or phosphate-buffered saline (n = 5) daily starting on day −7 before inoculation with bacteria and continuing until the animals were sacrificed on day 10 post-infection. Cumulative bone destruction scores (**A**) and frequency of bone destruction (**B**) of *P. aeruginosa* infected NMRI mice on day 10 post-infection as assessed by micro-computed tomography scan. A representative micro-computed tomography image (**C**) of an intact knee from healthy NMRI mouse treated with PBS (left panel) and damaged knee joint from NMRI mouse with septic arthritis treated with IL-1Ra (right panel). The arrow indicates bone destruction. Statistical evaluations were performed using the Mann–Whitney U test (**A**) and Fisher’s exact test (**B**). Data are expressed as mean values ± SEM.

We have previously shown that treatment with IL-1 receptor antagonist (Ra) aggravates *S. aureus*-induced septic arthritis and sepsis in mice^8^. In the present study, we investigated the role of IL-1 in mice infected with *P. aeruginosa*. IL-1 Ra treatment tended to aggravate *P. aeruginosa* arthritis, but no significant difference regarding either the severity or the frequency of bone erosion was observed (Fig. 5A–C).

### Antibiotic treatment reverses bone erosion in mice

**Figure 6:**
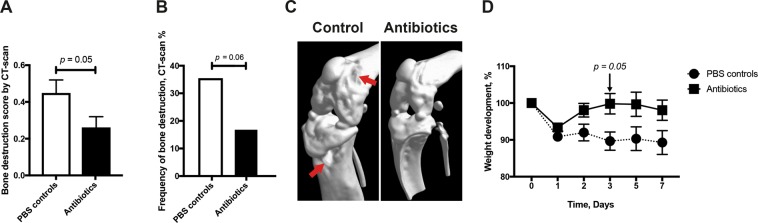
Antibiotic treatment prevents development of bone erosion in mice. Naval Medical Research Institute (NMRI) mice infected with *Pseudomonas aeruginosa* (*P. aeruginosa*) (5.6 × 10^7^ colony forming units [CFU]/mouse) were intraperitoneally treated with a volume of 300 μl Ciprofloxacin Villerton (2 mg/ml) twice daily starting from day 1 post-infection until termination of the experiment on day 7 post-infection. The same volume of PBS served as controls (n = 4/group). Cumulative bone destruction scores (**A**) and frequency of bone destruction (**B**) of *P. aeruginosa* infected NMRI mice on day 7 post-infection as assessed by micro-computed tomography scan. A representative micro-computed tomography image (**C**) of a destroyed knee from NMRI mouse with septic arthritis treated with PBS (left panel) and an intact healthy knee joint from NMRI mouse treated with antibiotics (right panel). The arrows indicate bone destruction. Changes in body weight (**D**) expressed as percentages of the initial body weight were also assessed. Statistical evaluations were performed using the Mann–Whitney U test (**A,D**), Fisher’s exact test (**B**).

We investigated if the development of bone erosion in *P. aeruginosa*-induced septic arthritis could be prevented with appropriate antibiotic treatment. Indeed, antibiotic treated mice had less severe arthritis (*p* = 0.05) as well as tendency toward lower frequency (*p* = 0.06) of bone erosion compared to PBS control mice (Fig. 6A–C).

Weight loss is an important parameter in our animal model that provides indication of the systemic effect of the disease. Here, we observed that PBS-treated control mice lost more weight (*p* = 0.05) compared to antibiotic-treated mice on day 3 post-infection (Fig. 6D).

### Treatment with antibiotics rescues mice from *P. aeruginosa*-induced death

As shown in Fig. 1, mice receiving higher dose of *P. aeruginosa* developed sepsis that led to death during the course of the experiment. We investigated if treatment with antibiotics could reverse this effect. Indeed, PBS-treated mice succumbed to *P. aeruginosa* infection within few days after infection whereas antibiotic-treated mice had significantly lower mortality (*p* = 0.003) (see Supplementary Fig. S2).

## Discussion

*S. aureus* remains the most common cause of septic arthritis. In comparison to *S. aureus*, *P. aeruginosa* is somewhat under-reported, but still one of the most common Gram-negative bacteria causing septic arthritis, especially in intravenous drug abusers^3^. Interestingly, despite similar surgical interventions and antibiotic treatment, patients with septic arthritis caused by *P. aeruginosa* had a lower remission rate than those infected with *S. aureus*, suggesting that *P. aeruginosa* is at least as clinically virulent as *S. aureus*^14^. The use of a unique animal model for *S. aureus* septic arthritis developed by our laboratory has clarified the involvement of several bacterial virulence factors, as well as the roles of various host immune cell types and cytokines involved in the pathogenesis of the disease^8–12^. However, there is no available animal model for *P. aeruginosa* septic arthritis. In the current study, we have established a novel mouse model for *P. aeruginosa* septic arthritis and demonstrated that neutrophils play a role in protection against disease. This model will also be useful to identify the virulence factors of *P. aeruginosa* in future studies.

Optimal arthritogenic dose is relatively narrow for the clinical *P. aeruginosa* strain used in this study, as the dose of 2.8 × 10^8^ CFU/mouse induced more than 50% mortality and 2.2 × 10^6^ CFU/mouse caused almost no signs of septic arthritis. Thus, the dose for different laboratory and clinical strains should be titrated and tested in the model. Our next step is to find the arthritis dose for the most used *P. aeruginosa* strains such as PA01, whose complete genome sequence has been reported^15^ and a library with large numbers of mutants are available to study the roles of different virulence factors in *P. aeruginosa* septic arthritis. The doses chosen should also be adjusted according to the purpose of the study or hypothesis in the different experiments. For example, to understand the role of neutrophils in *P. aeruginosa* arthritis, the bacterial dose should be kept at a lower level, since marked down-regulation of innate immunity by neutrophil depletion leads to uncontrolled systemic infection and death, which was the case in our study.

Pro-inflammatory cytokines are an essential part of the immune response required to eliminate invading microorganisms. Nevertheless, high levels of TNF-α and IL-6 have been shown to aggravate joint destruction in septic arthritis^16,17^ and anti-TNF therapy is known to ameliorate *S. aureus* septic arthritis^18^. In the current study, significantly higher levels of TNF-α and IL-6 were observed in supernatants of joint homogenates collected from *P. aeruginosa* infected mice compared to supernatants from healthy mice.

Some elements of innate immunity, including neutrophils^19^, complement factors^11^ and natural killer cells^20^, are protective in *S. aureus* septic arthritis. On the other hand, monocytes/macrophages are pathogenic in arthritic lesions, but protective in septic lethality^21^. In agreement with the data from *S. aureus*-induced septic arthritis, our current study compellingly demonstrates that neutrophils are the most crucial immune cells for better survival rates and for less severe septic arthritis, in our model of *P. aeruginosa*-induced septic arthritis. Indeed, both neutropenic mice and MyD88 deficient mice that cannot recruit neutrophils to lungs are highly susceptible to fatal *P. aeruginosa* lung infection^22^. CXC chemokines are critical mediators of neutrophil-mediated host defence in *P. aeruginosa* pneumonia^23,24^. Interestingly, *P. aeruginosa* itself possesses immune evasion capacity by producing ExoS and ExoT, two type III secreted effectors, blocking reactive oxygen species production by neutrophils^25^.

Remarkably, in mice with chemotherapy-induced neutropenia, vaccine-induced lung macrophage expansion protects against lethal *P. aeruginosa* pneumonia^26^, suggesting the potent role of monocytes/macrophages in *Pseudomonas* infections. In mice with septic arthritis caused by *S. aureus*, depletion of mononuclear phagocytes by etoposide led to deteriorated bacterial clearance and higher mortality^21^. The severity of arthritic lesions was, however, less pronounced in macrophage-depleted mice, suggesting a pathogenic role of macrophages in the development of septic arthritis. In the current study, monocyte/macrophage depletion by administering clodronate liposomes significantly increased mortality, which is in line with previous findings in *S. aureus* septic arthritis^21^. However, the protective effect of monocytes/macrophages depletion was totally absent in our animal model, as monocyte-depleted mice had actually slightly more severe bone erosions, demonstrating that macrophages are not pathogenic in *P. aeruginosa*-induced septic arthritis.

CD4+ T lymphocytes constitute various T helper cell subsets including Th1, Th2, Th17, Tfh and Tregs. Within CD4+ subsets, there is a large proportion of inflammatory cells that contribute to autoimmune and infectious diseases^27^. CD4+ T cells are known to be directly involved in the development of autoimmune arthritis^27^, *S. aureus* septic arthritis^13^ and Lyme arthritis^28^. Similarly, depletion of CD4+ but not CD8+ cells significantly reduced the severity and frequency of bone erosions in *P. aeruginosa*-induced septic arthritis, strongly indicating that CD4+ T cells are pathogenic in this disease. Which subsets of CD4+ T cells are responsible for this? Interferon-gamma (IFN-γ) producing Th1 cells are considered to be a major player in development of rheumatoid arthritis^29^. IFN-γ released by Th1 cells activates macrophages to produce pro-inflammatory cytokines such as TNF^30^. Th17 cells, another subset of CD4+ T cells, appear to play a potent role in the onset of autoimmune arthritis in several experimental models^31,32^. IL-17 blocking agents have been successfully used in psoriatic arthritis and ankylosing spondylitis^33,34^. Therefore, Th1 and Th17 are most likely the culprits, although further studies are needed to elucidate more detailed mechanisms.

MCP-1 is crucial for recruiting monocytes, neutrophils, as well as T-cells, to the site of an infection^35,36^. However, MCP-1 has been implicated in the pathogenesis of several diseases, e.g., rheumatoid arthritis^37^. Furthermore, MCP-1 has been shown to be upregulated in *P. aeruginosa* corneal infection in mice^38^ and treatment with anti MCP-1 antibodies resulted in significant reductions in severity of corneal damage and neutrophil infiltration^39^. Indeed, MCP-1 serum levels were elevated in mice with *P. aeruginosa* septic arthritis compared to healthy controls, suggesting that MCP-1 is implicated in *P. aeruginosa* septic arthritis. However, the role of MCP-1 in this disease might be much more complicated, since mice depleted of CD4+ T-cells displayed less severe bone destruction but higher MCP-1 serum levels compared to control animals.

IL-1β is produced predominantly by macrophages and plays potent roles in the early recruitment of neutrophils and subsequent bacterial killing in *P. aeruginosa* pulmonary infection^40,41^. IL-1Ra treatment has recently been shown to aggravate *S. aureus* septic arthritis^8^. Similarly, in the current study, IL-1Ra treatment showed a tendency towards enhanced severity and frequency of bone erosions caused by *P. aeruginosa* infection although statistically significant differences in this study could not be reached, probably due to small sample size.

In summary, we demonstrate that we have successfully established a mouse model for *P. aeruginosa* induced septic arthritis. Our results strongly suggest that neutrophils are protective for both septic arthritis as well as *P. aeruginosa* induced mortality. However, monocytes/macrophages are protective against *P. aeruginosa* induced death but exhibited no role against septic arthritis. CD4+ T cells play a pathogenic role in septic arthritis. Our model system is useful, not only to understand the pathogenesis of *P. aeruginosa* septic arthritis, but also to study virulence factors of *P. aeruginosa*.

## Methods

### Ethics statement

All animal experiments were approved by the Ethics Committee of Animal Research of Gothenburg and conducted in accordance with recommendations from the Swedish Board of Agriculture.

### Mice

Female 6–8 weeks old NMRI (Naval Medical Research Institute) mice were purchased from Envigo (Venray, Netherlands) and housed under standard conditions of temperature, nutrition and light in the animal facility of the Department of Rheumatology and Inflammation Research, University of Gothenburg.

### Preparation of bacterial solutions

*P. aeruginosa* strain CCUG 551 T (=ATCC 10145 T), the type reference strain of the species, (provided by Culture Collection of the University of Gothenburg, CCUG; www.ccug.se), was used in all the experiments. The bacterial suspensions were thawed, washed in phosphate-buffered saline (PBS), and adjusted to the concentration required before conducting the experiments.

### Experimental protocols for *P. aeruginosa* septic arthritis

Several separate *in vivo* experiments were performed for the *P. aeruginosa* infection studies. The mice received 200 μl of *P. aeruginosa* suspension i.v. into the tail vein in all the experiments. At the termination of the experiments, the mice were anaesthetized with medetomidine (Orion Pharma, Finland) and ketamine hydrochloride (Pfizer AB, Sweden), blood from the axillary artery was collected and the mice were immediately sacrificed as previously described^8^.

The experiments were performed as follows: (1) mice (n = 5/group) were inoculated with 4 different doses of *P. aeruginosa* ranging from 2.2 × 10^6^–2.8 × 10^8^ CFU/mouse to study the dose-dependent kinetics of arthritis; 2) mice (n = 9–10/group) were depleted of either neutrophils or macrophages and infected with *P. aeruginosa* (2.0–5.6 × 10^6^ CFU/mouse) to study the roles of neutrophils and macrophages in *P. aeruginosa-*induced septic arthritis; (3) mice (n = 5/group), depleted of either CD4 or CD8 T-cells, were infected with *P. aeruginosa* (1.2 × 10^7^ CFU/mouse), in order to investigate the impact of T-cells in *P. aeruginosa* septic arthritis; (4) mice (n = 5/group) treated with IL-1 Ra were inoculated with *P. aeruginosa* (2 × 10^6^ CFU/mouse) to study the role of IL-1 in septic arthritis; (5) mice (n = 4/group) were infected with *P. aeruginosa* (5.6 × 10^7^ CFU/mouse) and treated with PBS or ciprofloxacin to investigate if treatment could prevent the development of bone destruction caused by *P. aeruginosa* in mice; (6) mice (n = 7–8/group) were infected with *P. aeruginosa* (1.7 × 10^8^ CFU/mouse) and treated with PBS or ciprofloxacin to investigate if treatment could protect septic lethality in mice.

Two observers blinded to the treatment groups regularly weighed and clinically examined the mice for incidences and severity of arthritis. The mice were sacrificed on days 9–10 and sera were collected to assess the cytokine levels. In addition, the kidneys and the paws were obtained for assessment of bacterial counts and radiological examination of bone erosions, respectively.

To study whether the bacteria invaded and exist in the joints, mice (n = 10) were inoculated with *P. aeruginosa* (7 × 10^7^ CFU/mouse), and different joint groups (forepaws and wrists, elbows, shoulders, back paws and ankles, knees, and hips) from each animal were collected separately and homogenized for CFU counts on day 7 when the clinical arthritis became evident.

### *In vivo* cell depletion procedures

#### Monocyte/macrophage depletion

For selective elimination of macrophages^42,43^, 200 μl of clodronate liposomes (Liposoma BV, Netherlands) were injected i.v. per mouse on day −1, before infection with *P. aeruginosa*, and on day 1, post-infection. The control mice were treated with PBS liposomes (Liposoma BV, Netherlands).

#### Neutrophil depletion

For selective depletion of murine blood neutrophils^44^, 400 μg of specific monoclonal anti-Ly6G antibody (clone 1A8; BioXCell) in 200 μl of PBS was intraperitoneally (i.p.) injected on day −1, before infection with *P. aeruginosa*, and on day 1, post-infection. The control mice were treated with isotype control antibody (clone 2A3; BioXCell).

#### T cell depletion

The T-cell depletion procedures were carried out as described before^45^. For selective depletion of CD4 T-cells, a specific rat anti-mouse CD4 monoclonal antibody (clone GK1.5; BioXCell) was used, whereas to selectively deplete CD-8 T-cells in mice, a rat anti-mouse CD8α (clone 2.43; BioXCell) monoclonal antibody was used. The control mice for both groups were treated with a rat IgG2b isotype control (clone LTF-2; BioXCell) monoclonal antibody. Mice received i.p. a dose of 400 μg/mouse/antibody in 200 μl of PBS on day −1, before infection with *P. aeruginosa*, and on days 3 and 7, post-infection.

#### Measurement of depleted cells by flow cytometry

The experimental protocols investigating the success rate of the cellular depletions mentioned above are described elsewhere^45^. The depletion efficacies for the targeted cells as analysed by flow cytometry were as follows: neutrophils (99.6%), monocytes (85.6%), CD4 T-cells (97.3%) and CD8 T-cells (90%)^45^.

#### Treatment with IL-1 receptor antagonist

In order to study the effect of IL-1 in *P. aeruginosa*-induced septic arthritis, Kineret® (Anakinra; Amgen), IL-1 Ra, was used, that has previously been shown to block biological function of murine IL-1^8,46^. Anakinra (400 μg/mouse in 100 μl of PBS) was administered subcutaneously daily for one week prior to infection of the mice with *P. aeruginosa*. The mice were treated daily until day 10 post-infection. PBS served as control.

#### Antibiotics treatment

To test if antibiotic treatment could reverse the negative consequences of *P. aeruginosa* on our septic arthritis model, mice were treated with ciprofloxacin, which has previously been shown to be effective against *P. aeruginosa* infection in murine model^47^. Mice were i.p. injected with 0.3 ml of Ciprofloxacin Villerton (Mylan Hospital), 2 mg/ml, twice daily starting from day 1 post-infection until termination of the experiment. PBS served as controls.

### Clinical arthritis evaluation

Observers blinded to the treatment groups visually examined the presence of arthritis in all four limbs of each mouse. Arthritis was defined as erythema and/or swelling of the joints. To evaluate the severity of arthritis, a clinical scoring system ranging from 0–3 was used, as previously described^8,48^.

### Bacteriologic examination

The kidneys from the mice were aseptically collected and observers blinded to the treatment groups assessed abscess formation; a scoring system ranging from 0–3 was used, as previously described^8^. Afterwards, the kidneys were homogenized, plated on blood agar plates, and quantified as CFUs.

### Radiological evaluation of arthritis by micro-CT

The mice were sacrificed, the joints removed, fixed in 4% paraformaldehyde for 3 days and transferred to PBS for 24 hours. Thereafter, all limb joints were scanned with Skyscan1176 micro-CT (Bruker, Antwerp, Belgium) as previously described^8,9,49^. The projection images were reconstructed into three-dimensional images using NRECON software (version 1.6.9.8; Bruker) and analyzed with CT-Analyzer (version 2.7.0; Bruker). After reconstruction, experienced observers (A.A and T.J) evaluated, in a blinded manner, the extent of bone and cartilage destruction on a grading scale from 0–3, as previously described^8,9,45,49^.

### Histopathological evaluation of arthritis

After the scanning, representative joints were decalcified, embedded in paraffin and sectioned with microtome. Tissue sections were thereafter stained with haematoxylin and eosin, as previously described^45,49^.

### Homogenate preparation and bacteriologic examination

The different joint groups were aseptically removed, homogenized with an Ultra Turrax T25 homogenizer (IKA, Staufen, Germany), diluted in PBS, spread on horse blood agar plates, and incubated for 24 hours at 37 °C, as previously described^8,45^. Viable counts of bacteria were performed and quantified as CFUs. A cutoff-point of 10 CFUs was applied, joints with more than 10 CFUs were considered positive. The homogenates were centrifuged at 13,000 rpm for 10 minutes and the supernatants were collected for cytokine analysis.

### Measurement of cytokine and chemokine levels

DuoSet ELISA Kits (R&D Systems, Abingdon, UK) were used to quantify the levels of chemokine monocyte chemoattractant protein (MCP-1) and cytokines tumor necrosis factor alpha (TNF-α), interleukin 6 (IL-6) and interleukin 10 (IL-10) in supernatants from knee joint homogenates, as well as in serum.

### Statistical analysis

For statistical analysis, GraphPad Prism version 7.0b software for Mac (GraphPad software, La Jolla, CA, USA) was used. To assess statistical significances, the Mann-Whitney U test, Fischer’s exact test, and Mantel Cox log-rank test, as appropriate, were used. Results are reported as the mean values ± standard error of the mean (SEM), unless indicated otherwise. A *p* value < 0.05 was considered statistically significant.

## Supplementary information

Supplementary file

## Data Availability

The datasets generated during and/or analysed during the current study are available from the corresponding author on reasonable request.
