# Supplementary material for: A novel mouse model for septic arthritis induced by Pseudomonas aeruginosa
Source: Sci Rep. 2019 Nov 14;9:16868. doi: 10.1038/s41598-019-53434-5 (PMC6856550; doi:10.1038/s41598-019-53434-5)
Supplement: Supplementary file 1 — Supplementary file [file 41598_2019_53434_MOESM1_ESM.pdf]

# Supplementary Information

# A novel mouse model for septic arthritis induced by *Pseudomonas aeruginosa*

Tao Jin[1,2], Majd Mohammad[1], Zhicheng Hu[1,3], Ying Fei[3], Edward R B Moore[4,5,6], Rille Pullerits[1,2,7], Abukar Ali[1*]

1. Department of Rheumatology and Inflammation Research, Institute of Medicine, Sahlgrenska Academy at University of Gothenburg, Göteborg, Sweden

2. Department of Rheumatology, Sahlgrenska University Hospital, Sweden

3. Department of Microbiology and Immunology, The Affiliated Hospital of Guizhou Medical University, China

4. Department of Infectious Diseases, Institute of Biomedicine, Sahlgrenska Academy, University of Gothenburg, Göteborg, Sweden.

5. Culture Collection University of Gothenburg (CCUG), Sahlgrenska Academy, University of Gothenburg, Göteborg, Sweden.

6. Centre for Antibiotic Resistance Research (CARe), University of Gothenburg, Göteborg, Sweden.

7. Department of Clinical Immunology and Transfusion Medicine, Sahlgrenska University Hospital, Sweden

**Figure S1**

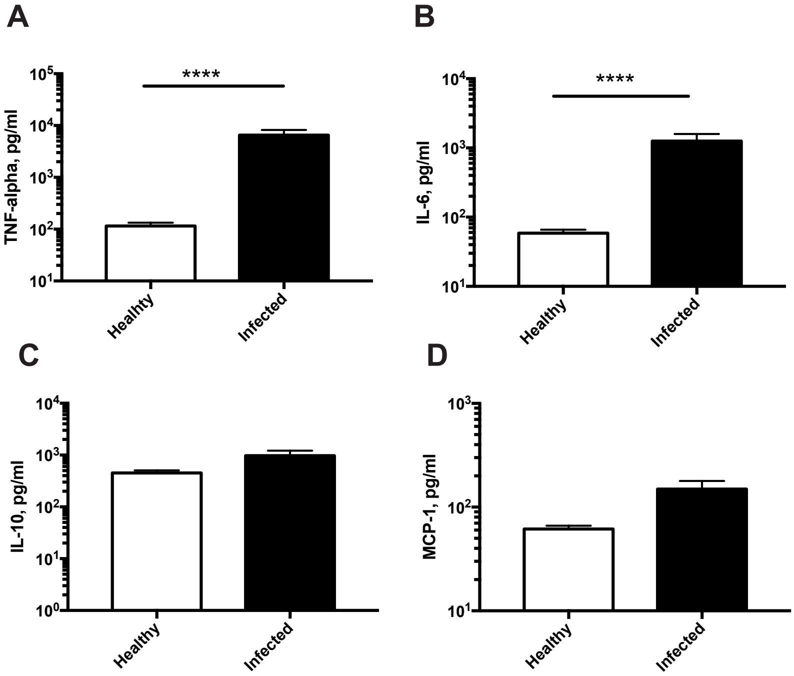


**Supplementary Figure S1. Elevated levels of TNF-α and IL-6 in joints of mice infected with *P. aeruginosa.*** The levels of tumor necrosis factor alpha (TNF-alpha) (**A**), interleukin 6 (IL-6) (**B**), interleukin 10 (IL-10) (**C**) and monocyte chemoattractantprotein 1 (MCP-1) (**D**) from supernatants collected from joint homogenates of *Pseudomonas aeruginosa* infected Naval Medical Research Institute (NMRI) mice (7 x $10^7$ CFU/mouse) were compared to supernatants from joint homogenates obtained from healthy mice (n=60-72 joints per group). Statistical evaluations were performed using the Mann–Whitney U test. Data are expressed as mean values ± SEM. **** = $p < 0.0001$

**Figure S2**

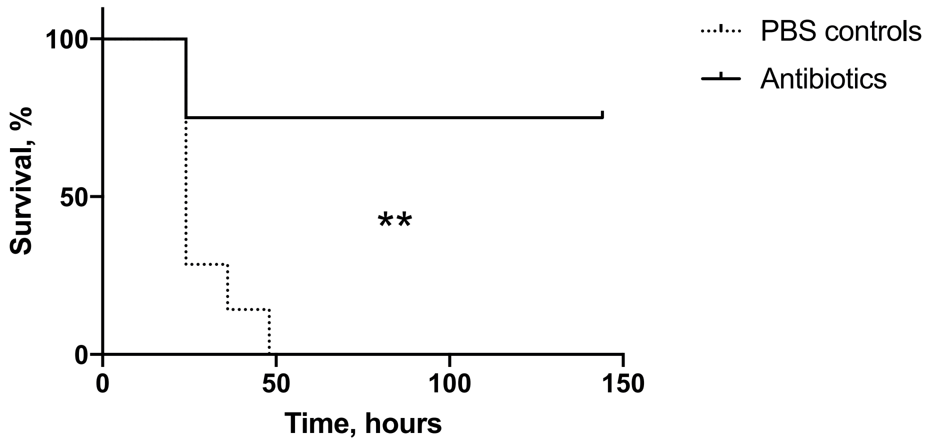


**Supplementary Figure S2. Treatment with antibiotics rescues mice from *P. aeruginosa*-induced death.**

Naval Medical Research Institute (NMRI) mice infected with *Pseudomonas aeruginosa* (1.7x$10^8$ colony forming units [CFU]/mouse) were intraperitoneally treated with a volume of 300 µl Ciprofloxacin Villerton (2mg/ml) twice daily starting from day 1 post-infection until termination of the experiment on day 7 post-infection. The same volume of PBS served as controls (n=7-8/group). The cumulative survival of the mice during the course of the experiment was assessed. Statistical evaluations were performed using the Log-rank Mantel cox. ** = $p < 0.01$.

**Supplementary Table S1**. Subgroup analyses of bone destructions by a micro-computed tomography scan.

| | Severity (Mean ± SEM) | | | |
|---|---|---|---|---|
| Bacterial dose (CFU/mouse) | $2.8x10^8$ | $5.6x10^7$ | $1.1x10^7$ | $2.2x10^6$ |
| Front paws | 0.8±0.8 | 0.1±0.1 | 0.2±0.2 | 0.0±0.0 |
| Hind paws | 1.5±0.6 | 0.6±0.2 | 0.4±0.2 | 0.25±0.1* |
| Elbows | 0.0±0.0 | 0.0±0.0 | 0.0±0.0 | 0.0±0.0 |
| Shoulders | 0.6±0.5 | 0.0±0.0† | 0.0±0.0† | 0.0±0.0 |
| Knees | 0.5±0.5 | 0.6±0.3 | 0.5±0.3 | 0.0±0.0 |
| Hips | 0.0±0.0 | 0.2±0.1 | 0.1±0.1 | 0.0±0.0 |

Statistical evaluations were performed using the Mann–Whitney *U* test. Data were presented as mean values ± standard error of the mean. * = $p < 0.05$ versus $2.8x10^8$; † $p$=0.06 versus $2.8x10^8$.

**Supplementary Table S2**. Subgroup analyses of bone destructions by a micro-computed tomography scan.

| | Frequency (%) | | | |
|---|---|---|---|---|
| Bacterial dose (CFU/mouse) | $2.8x10^8$ | $5.6x10^7$ | $1.1x10^7$ | $2.2x10^6$ |
| Front paws | 25 | 10 | 10 | 0 |
| Hind paws | 75 | 44 | 30 | 20 |
| Elbows | 0 | 0 | 0 | 0 |
| Shoulders | 25 | 0 | 0 | 0 |
| Knees | 25 | 30 | 20 | 0 |
| Hips | 0 | 10 | 0 | 0 |

Statistical evaluations were performed using the Fisher's exact test.

**Supplementary Table S3**. Subgroup analyses of bone destructions by a micro-computed tomography scan.

| | Severity (Mean ± SEM) | | Frequency (%) | |
|---|---|---|---|---|
| | Isotype controls | Anti-Ly6G | Isotype controls | Anti-Ly6G |
| Front paws | 0.0±0.0 | 0.0±0.0 | 0 | 0 |
| Hind paws | 0.2±0.1 | 0.0±0.0 | 10 | 0 |
| Elbows | 0.0±0.0 | 0.3±0.3 | 0 | 17 |
| Shoulders | 0.0±0.0 | 0.3±0.3 | 0 | 17 |
| Knees | 0.0±0.0 | 1.0±0.6* | 0 | 33* |
| Hips | 0.0±0.0 | 0.5±0.5 | 0 | 17 |

Statistical evaluations were performed using the Mann–Whitney U test or Fisher's exact test. Data were presented as mean values ± standard error of the mean. * = $p < 0.05$.

**Supplementary Table S4**. Subgroup analyses of bone destructions by a micro-computed tomography scan.

| | Severity (Mean ± SEM) | | Frequency (%) | |
|---|---|---|---|---|
| | PBS control liposomes | Clodronate liposomes | PBS control liposomes | Clodronate liposomes |
| Front paws | 0.1±0.1 | 0.0±0.0 | 11 | 0 |
| Hind paws | 0.1±0.1 | 0.4±0.2 | 6 | 30 |
| Elbows | 0.0±0.0 | 0.0±0.0 | 0 | 0 |
| Shoulders | 0.0±0.0 | 0.0±0.0 | 0 | 0 |
| Knees | 0.1±0.1 | 0.0±0.0 | 6 | 0 |
| Hips | 0.0±0.0 | 0.0±0.0 | 0 | 0 |

Statistical evaluations were performed using the Mann–Whitney U test or Fisher's exact test. Data were presented as mean values ± standard error of the mean.

**Supplementary Table S5**. Subgroup analyses of bone destructions by a micro-computed tomography scan.

| | Severity (Mean ± SEM) | | | Frequency (%) | | |
|---|---|---|---|---|---|---|
| | Isotype controls | anti-CD4 | anti-CD8 | Isotype controls | anti-CD4 | anti-CD8 |
| Front paws | 0.2±0.1 | 0.1±0.1 | 0.3±0.2 | 10 | 10 | 30 |
| Hind paws | 0.4±0.2 | 0.6±0.3 | 0.0±0.0 | 20 | 40 | 0 |
| Elbows | 0.1±0.1 | 0.0±0.0 | 0.0±0.0 | 10 | 0 | 0 |
| Shoulders | 0.3±0.2 | 0.0±0.0 | 0.6±0.3 | 10 | 0 | 20 |
| Knees | 1.4±0.4 | 0.0±0.0* | 1.3±0.4 | 60 | 0* | 40 |
| Hips | 0.3±0.3 | 0.0±0.0 | 0.4±0.4 | 10 | 0 | 12.5 |

Statistical evaluations were performed using the Mann–Whitney U test or Fisher's exact test. Data were presented as mean values ± standard error of the mean. * = $p < 0.05$.
